# Development of a Decision Aid to Support Shared Decision-Making on Cannabis Use for Arthritis: Protocol for a Multiphase Study

**DOI:** 10.2196/76237

**Published:** 2026-03-30

**Authors:** Heba A T Aref, Yazid N Al Hamarneh, Tony K L Kiang, Elaine Yacyshyn, Cheryl A Sadowski

**Affiliations:** 1 Faculty of Pharmacy & Pharmaceutical Sciences, University of Alberta, 3-229 Dianne and Irving Kipnes Health Research Academy, 11405 87 Ave. NW, Edmonton, AB, T6W 0B3, Canada, 1 780-492-5078, 1 780-492-1217; 2 Department of Pharmacology, Faculty of Medicine and Dentistry, University of Alberta, Edmonton, AB, Canada; 3 Department of Medicine, Faculty of Medicine and Dentistry, University of Alberta, Edmonton, AB, Canada

**Keywords:** decision support intervention, cannabis, shared decision-making, informed decision-making, decision aid

## Abstract

**Background:**

Conventional treatments have been frequently reported to offer partial relief for some individuals managing arthritis pain and related symptoms, leading many to consider alternative options, such as cannabis. Informed decision-making about cannabis use requires patients to weigh potential benefits and risks in light of their personal values and preferences.

**Objective:**

This study aims to systematically develop a theory-driven, evidence-based, user-centered decision aid (DA) for older adults with arthritis, considering medical cannabis.

**Methods:**

The Ottawa Decision Support Framework, the International Patient Decision Aids Standards, and the User-Centeredness approach guided the DA development process in 4 phases. Phase 1 (completed) involved scoping reviews to explore the development of shared decision-making (SDM) tools and the literature addressing cannabis use in arthritis, guided by the PRISMA (Preferred Reporting Items for Systematic Reviews and Meta-Analyses) framework. Phase 2 (in progress) consists of semistructured interviews that are conducted with older adults living with arthritis to explore their decisional needs. Phase 3 (in progress) involves semistructured interviews that are conducted with clinicians to explore how they can participate with their patients during cannabis use decision-making. For phases 2 and 3, recruitment has been initiated in a large urban center in Alberta, Canada. Purposive sampling is conducted, and the sample size will be guided by the principle of information power. Phase 4 (not initiated yet) will involve the development of the DA and alpha testing to explore qualification as a DA and acceptability with advisory board members, including patients and clinicians who participate in the development process. Based on the feedback received, the prototype will be refined accordingly. Subsequently, a future study involving a beta testing phase will be conducted to evaluate the usability, accessibility, and comprehensibility of the DA among naive users. The University of Alberta Human Research Ethics Board approved this study. This protocol is based on the latest version of the ethics application, dated December 14, 2024 (Pro00133420).

**Results:**

This project was funded in November 2022. Phase 1 is completed; the advisory board is assembled. Two scoping reviews have been published. The first review showed that the most commonly reported development bases for SDM tools were the Ottawa Decision Support Framework and International Patient Decision Aids Standards criteria, reported in 16% and 53% of tools, respectively. The second review highlighted limited high-quality evidence addressing medical cannabis use for arthritis. Phases 2 and 3 have been initiated. Phase 4 will be initiated when the findings from phases 2 and 3 are available. As of November 2025, we have recruited 10 patients for phase 2 and 7 clinicians for phase 3.

**Conclusions:**

This protocol outlines the systematic development of a patient-centered DA to support SDM about medical cannabis for arthritis pain. The findings will show the feasibility of development and inform the refinement of the DA, guiding its readiness for beta testing and future implementation.

## Introduction

Approximately 1 in 3 adults diagnosed with arthritis diseases (eg, gout, rheumatoid arthritis, and osteoarthritis) have severe chronic joint pain that impacts their quality of life [1]. Many pharmacological agents (eg, nonsteroidal anti-inflammatory drugs, acetaminophen, and opioid analgesics) are available to address such pain [2,3]. However, unsatisfactory responses and adverse reactions have raised interest in using alternative therapies, such as medical cannabis [4-6]. Indeed, arthritic pain has been reported to be one of the most common indications for using medical cannabis in North America [7].

In Canada, patients with arthritis are increasingly inquiring about cannabis as an alternative treatment. This could be explained by the recent legalization of medical cannabis, the aggressive marketing of the cannabis industry, the slow social shift of the stigma of using cannabis for medical purposes, and medical research that reported a limited positive impact of cannabis within specific contexts [7-10]. Despite this increased curiosity, it has been reported that clinicians lack confidence in their knowledge about cannabis [11], as empirically established clinical evidence is inconsistent, and definitive cannabis use guidelines are not yet available [4,12-14]. As such, patients are currently making the decision to use cannabis based on individual preferences and values without having clear guidelines [15,16].

The current lack of cannabis use guidelines, combined with the complexity of cannabis-related decisions, highlights the need for utilizing shared decision-making (SDM) [17,18]. Specifically, patients and clinicians must navigate numerous uncertainties unique to medical cannabis, including (1) the wide variability in cannabinoid formulations, ratios, and delivery methods, each with different onset times and duration of effects [19]; (2) the absence of standardized dosing protocols and the need for individualized titration [15]; (3) potential drug-drug interactions, particularly concerning for older adults with polypharmacy [20]; and (4) weighing potential benefits such as pain reduction and improved sleep quality against risks including cognitive impairment, falls, and cardiovascular effects [21]. These complexities are further compounded by varying legal frameworks, insurance coverage limitations, and persistent stigma surrounding cannabis use [22-24]. Such multidimensional trade-offs and uncertainties make the cannabis use decision for arthritis particularly suited for SDM [25,26]. SDM is the communication process through which patients and clinicians work together collaboratively using specific tools to reach a treatment decision based on integrating clinical evidence with patients’ preferences, values, and goals [22]. SDM improves patients’ satisfaction and adherence and leads to a better quality of life [23,24]. However, SDM has not been widely incorporated into daily practice due to barriers related to the lack of decision support tools that facilitate SDM within a busy organizational workflow [25-27].

Decision aids (DAs) are structured decision support interventions (DSIs) that systematically present evidence and elicit patient values, especially in situations of preference-sensitive decisions [25,26], such as cannabis use. A DA is designed to present evidence-based information about available options, along with their potential benefits and harms, to support patients in making informed, deliberate, and value-aligned choices [25,27,28]. As such, DAs reduce uncertainty about decision-making and improve the quality of health care decisions, leading to better health outcomes [29,30]. Several DAs have been developed for arthritis diseases [18,31,32]; however, to our knowledge, none of them addressed the topic of cannabis use for patients with arthritis. The need for a DA to facilitate the decision of whether to use cannabis or not is evident, as there is a scarcity of strong recommendations, guidelines, or benefit-to-harm assessments addressing the effectiveness and safety of cannabis for arthritis pain and other symptoms.

Therefore, the objective of this study is to systematically develop a theory-driven, evidence-based, user-centered cannabis DA for older adults with arthritis, guided by the Ottawa Decision Support Framework (ODSF) and the International Patient Decision Aids Standards (IPDAS). We will evaluate the feasibility of developing the first prototype ready for beta testing. Feasibility will be determined through the completion of prespecified milestones, including evidence synthesis, decisional needs assessment, prototype development, iterative refinement through incorporating feedback from the patients and clinicians in the advisory board input, and full documentation following the IPDAS DEVELOPTOOLS reporting checklist [33].

## Methods

### Study Design and Conceptual Framework

This protocol outlines 4 key phases of development, as illustrated in Figure S1 in Multimedia Appendix 1, with a summary of procedures in Table S1 in Multimedia Appendix 2. The phases comprise phase 1: preliminary work, phase 2: qualitative descriptive study exploring patients’ decisional needs, phase 3: qualitative descriptive study exploring clinicians’ decisional needs, and phase 4: development and alpha testing. The study is guided conceptually by the ODSF [34,35]—a theory-based framework (grounded in decisional conflict theory [36] and self-efficacy theories [37])—and methodologically by the IPDAS development model [33,38,39].

### Outcome Measures

#### Primary Outcome

The primary outcome of this project is to demonstrate the feasibility of systematically developing a theory-driven, evidence-based DA for a context characterized by limited and evolving evidence—specifically, medical cannabis use for symptom management among older adults with arthritis. Feasibility will be demonstrated through (1), recruitment of at least 80% (n=8-10) of the target sample with ongoing consideration of information power, (2) retention of ≥80% (n=8-10) of participants recruited in phases 2 and 3 to provide feedback for phase 4, (3) adherence to a 12‐ to 18-month development timeline (based on comparable studies), (4) the generation of actionable user feedback to guide iterative refinement, and (5) comprehensive reporting of each development phase in accordance with the IPDAS-recommended DEVELOPTOOLS checklist [33]. Meeting these prespecified benchmarks will indicate the feasibility of developing a prototype ready to progress to beta testing.

#### Secondary Outcome

The secondary outcome is to obtain acceptability and qualification as data that support proceeding to the beta testing phase. We will conduct the acceptability [40] and the DA qualification assessment [41] as part of alpha testing (iteration with participants). These assessments will provide structured feedback for iterative formative refinement rather than definitive validation for implementation. Measures and scoring procedures are prespecified under phase 4b. This approach will identify aspects requiring refinement before beta testing with naive users and is consistent with iterative development processes for DAs [33,38].

### Phase 1: Preliminary Work (Completed)

#### Phase 1a: Assembling the Advisory Board

The advisory board was assembled under the leadership of the principal investigators, CAS (geriatrics specialized pharmacist) and EY (rheumatologist), who served as the first 2 members of the board. Drawing on their professional networks, they invited members with relevant expertise. Common interests were verified through conversations to ensure a collaborative, multidisciplinary team committed to supporting the development of a DA. The advisory board defined the scope and purpose of the DA.

#### Phase 1b: Scoping Reviews

Two reviews were conducted to scope the literature on (1) DSIs among patients with arthritis and (2) cannabis use in arthritis [42,43]. Both studies used systematic search and evidence synthesis approaches guided by Arksey and O’Malley’s methodological framework and the PRISMA-ScR (Preferred Reporting Items for Systematic reviews and Meta-Analyses extension for Scoping Reviews) checklist [44,45].

### Phases 2 and 3: Two Qualitative Studies (in Progress)

#### Overview

Phases 2 and 3 aimed to explore patients’ and clinicians’ perspectives on cannabis use decision-making. Figure S2 in Multimedia Appendix 3 shows the participant flow throughout the study.

We adopted a qualitative descriptive approach, an inquiry method aiming at providing a comprehensive summary of participants, staying close to the surface of the data without extensive interpretation or theorizing [46-48]. This approach is particularly well suited for exploring the decisional needs of patients and clinicians, as it allows for the direct capture of participants’ perspectives in their own words—an essential consideration when the goal is to inform the development of practical, user-centered DA.

#### Positionality Statement

The research team acknowledges that their professional backgrounds and clinical experiences will inevitably shape how participants’ statements are interpreted. The lead researcher (HATA) is a health researcher with training in quantitative and qualitative methodologies and experience in decision-making, geriatrics, and chronic disease management, which may be associated with increased awareness of issues related to medical cannabis safety and evidence uncertainty. Coauthors’ stance aligns with the Canadian Rheumatology Association (CRA) Position Statement recognizing insufficient evidence for medical cannabis in arthritis, while calling for empathetic, pragmatic guidance that supports respectful patient-clinician dialogue and prioritizes harm reduction [49]. This stance may predispose the team toward cautious, harm reduction-oriented interpretations of participants’ experiences. To address this, we will engage in ongoing reflexive practice, including memoing and analytic discussions, to critically examine how our clinical perspective may influence theme development and framing.

#### Data Collection

For phase 2, the target population will comprise patient participants who are 55 years of age or older living with any form of arthritis, currently using, interested in, considering, or declining medical cannabis for symptom management. We initiated recruitment in a large urban center in Alberta, Canada. For phase 3, the target population will comprise clinician participants (eg, pharmacists, rheumatologists, family physicians, and nurse practitioners) involved in arthritis care and counseling about symptom management options. We initiated recruitment through advertising in a local medical newsletter.

Purposive sampling will be used, and the sample size will be guided by the principle of information power, wherein the adequacy of the sample depends on the degree to which participants collectively contribute information relevant to the study aim [50]. This approach is consistent with reflexive thematic analysis (RTA), which does not rely on saturation as a marker of adequacy [51,52]. High information power [50] is anticipated, given the narrow research aim (decisional needs regarding medical cannabis for arthritis), the high specificity of the sample, the use of an established theoretical framework (the ODSF) [30], and the expected richness of semistructured interviews guided by a decisional needs assessment workbook [53]. Based on comparable decisional needs studies, we anticipate recruiting approximately 10 to 12 participants per phase. The research team will review data relevant to ODSF constructs and assess information power after every 3 interviews to determine whether additional recruitment is required [50]. Recruitment will cease once consensus among advisory board members indicates that the accumulated data provide sufficient depth, breadth, and relevance to meet the study aim. The inclusion criteria are given in Textbox 1. Eligible participants who agree to participate will be provided with an information letter and consent form outlining the study’s purpose, procedures, participants’ rights, confidentiality and anonymity protocols, and data storage practices. A trained researcher (HATA) will conduct all one-on-one semistructured interviews using interview guides for patients and clinicians (MultimediaAppendices 4  5) informed by the decisional needs assessment approach, the ODSF, and probing techniques [34,53-55]. To support accessibility and accommodate participants’ preferences, interviews will be conducted virtually via a secure video conferencing platform, with telephone or in-person options available as needed. All interviews will be audio-recorded with participant consent and are expected to last approximately 45 to 60 minutes. At the end of each interview, patients and clinicians will be asked to provide demographic information (eg, for patients: age, education level, and cannabis usage status and for clinicians: years of practice, age, and area of practice). Audio recordings will be transcribed verbatim using Quirkos [56] and deidentified using unique participant codes (eg, P01 for patients, C01 for clinicians). Transcripts will be cross-checked against audio recordings for accuracy and completeness. Transcripts, audio files, and the corresponding linking key will then be securely stored on encrypted, password-protected university servers accessible only to the research team and compliant with institutional data protection policies.

Textbox 1. **Inclusion criteria**

**Phase 2 (patients)**

- 55 years of age or older (although the World Health Organization generally defines older adults as those aged 60 years and older, chronic conditions, such as arthritis, increase from the mid-50s onward [5,57,58]. For this study, “older adults” are individuals aged 55 years or older, consistent with aging and arthritis research identifying 55 years of age as the onset of heightened chronic disease burden [5,57-61]
- Living with arthritis
- Considering, currently using, have previously used, or have explicitly decided against using cannabis for arthritis symptom management

**Phase 3 (clinicians)**

- Currently treating patients with arthritis
- Have experience supporting or making decisions about cannabis use (eg, responding to patient questions about cannabis, recommending to use or avoid cannabis)
- Interested in describing their needs to participate with patients in making a decision about cannabis use

#### Data Analysis

To ensure transparency, rigor, and methodological clarity, both qualitative studies will be reported in accordance with the Standards for Reporting Qualitative Research guidelines [62]. The data from both phases will be analyzed using RTA situated within a qualitative descriptive approach, an analytic combination that enables the researcher to remain close to participants’ accounts while acknowledging that meaning-making is inherently interpretive [46-48,51]. RTA provides methodological flexibility suitable for exploring decisional needs in depth, while the qualitative descriptive approach ensures that findings are grounded in the language and experiences of older adults and clinicians [48,51].

A single researcher (HATA) will lead all analytic activities, consistent with RTA’s epistemological stance that positions the researcher as the primary analytic instrument and supports sole-coder analysis as a flexible, coherent, and methodologically appropriate practice [51,63]. The analysis procedure will begin with familiarization through repeated reading of verbatim transcripts and verification of transcription accuracy to immerse oneself in the data. A hybrid deductive–inductive approach will be used for coding. An initial coding framework based on the ODSF will be applied deductively to identify predefined decisional needs constructs (eg, knowledge gaps, expectations, values, decisional conflict, and support needs). In parallel, inductive coding will be used to capture unanticipated themes emerging from participants’ accounts and the cannabis context. Codes will be iteratively refined and organized into higher-order themes through RTA. Final themes will be explicitly mapped to the DA components, including informational content, values-clarification exercises, and clinician-patient discussion prompts, ensuring that the DA content is directly informed by empirically identified decisional needs of both patients and clinicians. Coding will be conducted in Quirkos [56] to organize excerpts, identify patterned meaning, and support early category development.

Throughout the analysis, HATA will generate initial themes, refining them through iterative cycles of reviewing and returning to the data, critical reflection, and agreement among the research team. This approach will serve to deepen interpretive insight by incorporating the research team’s diverse disciplinary standpoints in health services research, geriatric pharmacotherapy, rheumatology, and pharmaceutical sciences, consistent with RTA’s emphasis on reflexivity over reliability [51]. Themes will be assessed for coherence, consistency, relevance, and alignment with the research aims and the goal of developing the DA. Finally, themes will be defined and named by refining their boundaries and ensuring clear definitions.

To ensure trustworthiness across both qualitative descriptive studies, multiple strategies will be used to establish credibility, dependability, confirmability, and transferability [64,65]. Credibility will be enhanced through advisory board review of thematic findings, negative-case analysis to identify divergent patterns, and triangulation across patient and clinician datasets to explore convergent, divergent, and complementary insights [66-68]. A comprehensive audit trail with a versioned codebook documenting analytic decisions and coding iterations will be maintained throughout analysis to transparently track coding evolution and methodological reflections supporting dependability, while confirmability will be supported through extensive reflexivity practices, including researcher journaling and reflexive memoing [69-71]. Transferability will be established through thick description of study context, procedures, and participant characteristics, enabling readers to assess applicability to other settings [72]. The advisory board will provide a final review of the thematic structure to evaluate its relevance and usefulness for informing the DA prototype development.

### Phase 4: Developing the DA (Not Initiated Yet)

#### Overview

Phase 4a focuses on generating the initial prototype, while phase 4b constitutes a formative, embedded alpha-testing phase situated within development rather than a summative validation intended for implementation. The purpose of alpha testing is to obtain structured, actionable feedback to refine the prototype prior to subsequent beta testing with naive users. To enhance methodological transparency and reproducibility, all outcome measures, instruments, scoring procedures, and a priori success thresholds are prespecified below.

#### Phase 4a: Developing the First Prototype of the DA

##### Overview

The development of the DA will follow a structured, transparent, and iterative process aligned with the IPDAS Collaboration criteria for content development, balance, and evidence reporting [73,74]. The prototype’s first draft will be developed based on phases 1-3, the IPDAS qualifying criteria [41], and recent cannabis evidence literature [43,75]. As it has been reported that DA development processes have been suboptimally reported [33,38], we plan to describe the development process comprehensively and transparently in a stand-alone paper. Witteman et al [33] highlighted 8 studies [32,76-84] as being exemplary in reporting their systematic approach. Therefore, we planned this phase based on these 8 studies, together with leveraging the DEVELOPTOOLS checklist that ensures a rigorous, transparent, systematic approach to DA development [33]. A structured content-governance process is established to ensure rigor and transparency as follows.

##### First: Cannabis Evidence Synthesis

Due to the current lack of a coherent and homogeneous body of clinical evidence and limited high-quality systematic reviews or clinical practice guidelines on medical cannabis use for arthritis, the current literature does not meet the methodological requirements needed to conduct a Cochrane systematic review or apply evidence-rating frameworks such as GRADE (Grading of Recommendations Assessment, Development, and Evaluation). Therefore, we will instead collect input from patients and clinicians on preferred evidence sources for content. In addition, the synthesis of evidence for content development will be informed by the findings from our team’s prior work, together with the updated position statement of the CRA [58]. Consistent with IPDAS guidance for emerging evidence contexts, uncertainty and evidence gaps will be transparently communicated throughout the DA.

##### Second: Balanced Content Development

To ensure a balanced presentation of potential benefits and harms, we will summarize evidence-based advantages, limitations, and risks of medical cannabis using the highest-quality evidence available at the time of development. In synthesizing evidence, we recognize that the findings from other jurisdictions may not be directly applicable to the Canadian context, where product availability and THC:CBD ratios and potencies differ markedly from settings, such as the United States, which operates under mixed state laws; lacks federal legalization; and exhibits substantial variability in product quality, access, and cannabinoid composition. Accordingly, Canadian data will be prioritized whenever possible, and international evidence will be interpreted cautiously. Evidence will be synthesized using balanced, nondirective, evidence-informed language that presents potential benefits and harms to support shared deliberation between patients and clinicians.

##### Third: Accessibility, Readability, and Numeracy Standards

Accessibility and readability standards will be considered during the development process and will be assessed later on during beta testing. The findings from phases 2 and 3 will determine patients’ and clinicians’ preferences for the format of the DA, whether a digital or paper-based. For either format, we plan to ensure that the DA is accessible and comprehensible for older adults. Therefore, we will adopt accessibility and readability standards consistent with health literacy best practices [38,85].

- Accessibility: The DA formats (digital or print) will conform to Web Content Accessibility Guidelines [86]. Features will include adjustable text size, high-contrast design, screen reader compatibility, alternative text for images, clear structure, and captions for all multimedia. For the print version, equivalent accessibility principles will be applied, including the use of large fonts (minimum 14 pt), high-contrast text and background colors, clear headings, sufficient white space, and simplified layout to support legibility for older adults.
- Readability: Content will be written in plain language at a Grade 6‐8 reading level, verified using the *Flesch–Kincaid Grade Level*. Short sentences, active voice, and simplified terminology will be prioritized. Visuals (icons and infographics) will be used to supplement text and enhance comprehension.
- Numeracy supports: Where feasible, quantitative findings will be reported in multiple formats (eg, consistent denominators, natural frequencies, visual displays) supplemented by plain-language explanations and visual aids to enhance comprehension across varying numeracy levels [87].

##### Fourth: Intervention Delivery

The delivery characteristics of the DA—including its format, timing, and the individuals involved in its delivery—will be determined based on the findings from phases 2 and 3. Grounding format decisions (digital vs nondigital) in qualitative insights further ensures that the DA accommodates the needs of older adult participants, consistent with standards for DA development. Following this, the DA will undergo further refinement during phase 4b, where advisory board feedback will be systematically integrated.

##### Fifth: Version Control and Update Cadence

A structured version-control protocol will be implemented to document all content iterations, authorship contributions, revisions, and editorial decisions. In accordance with IPDAS quality criteria, all research team and advisory board members will be required to disclose potential conflicts of interest, which will be reviewed during each update cycle. All funding sources will be transparently reported [88]. Evidence sources will be systematically cataloged and critically reappraised every 2 years, with provisions for earlier review should stronger evidence, regulatory changes, emerging safety considerations, or jurisdiction-specific policy updates (eg, Health Canada or CRA guidance) arise. This governance structure will help ensure that DA’s evidence summaries remain current, accurate, and aligned with evolving scientific and regulatory developments.

### Phase 4b: Alpha Testing the Prototype With the Advisory Board

Alpha testing will evaluate whether the DA is acceptable to individuals familiar with its development and whether it qualifies as a DA, following the iterative early-testing processes described in IPDAS development literature. Advisory board members will be asked to perform 2 tasks.

#### Task 1: Structured Assessment of DA Qualification and Acceptability

Each participant will independently review the DA and complete two questionnaires:

1. IPDAS Qualifying Criteria Questionnaire (Multimedia Appendix 6): This questionnaire will be used to evaluate adherence to core DAs standards, including articulation of the decision; identification of target users; balanced presentation of options; clarity regarding benefits and harms; inclusion of values-clarification components; and transparency of evidence sources, authorship, and funding [41]. The DA will be deemed ready for beta testing if ≥75% of participants report that all IPDAS qualifying criteria are met and confirm compliance with bias-reduction standards. Thus, advisory board members (patients and clinicians) will classify whether the prototype qualifies as a DA using the IPDAS qualifying criteria. To qualify, the prototype must meet all 8 essential “qualifying criteria” (eg, defining the decision, presenting options, providing balanced benefits and harms, including a values-clarification exercise) and satisfy 9 criteria that reduce the risk of biased decision-making (eg, balanced presentation, citation of evidence sources, disclosure of conflicts and funding) [41].
2. Acceptability questionnaire (Multimedia Appendix 7): This questionnaire will assess advisory board members’ perceptions of the DA’s clarity, comprehensibility, usability, neutrality, level of detail, usefulness for decision-making, compatibility with clinical workflow, and overall suitability for adoption in practice [40]. The DA will meet acceptability thresholds if: (1) ≥75% (n=7-9) of participants rate each informational component as “good” or “excellent,” (2) no component receives >25% (n=2-3) “poor” ratings, and (3) ≥75% of all questionnaire responses fall within “agree/strongly agree” (ie, mean score ≥4.0) [38,40,74].

#### Task 2: Semistructured Cognitive Interview

Think-aloud and semistructured cognitive interview will be conducted with all participants to contextualize and corroborate questionnaire results [33,38,89]. Participants will elaborate on reasons for high or low ratings; perceived ambiguities or biases; navigation challenges or areas of cognitive burden; and suggestions for improving clarity, neutrality, and integration into clinical encounters.

Interviews will be audio-recorded, and field notes will capture contextual observations. Interviews will be analyzed to identify usability concerns, refine content, and guide iterative improvements before beta testing.

Quantitative and qualitative findings will be analyzed and used together to identify issues to be addressed through iterative refinement. All changes will be logged in a structured decision log, aligning with DEVELOPTOOLS recommendations for transparent reporting [33].

This alpha-testing stage ensures that the DA is evidence-based, clinically accurate, and responsive to end user perspectives before beta testing. By integrating evidence synthesis, structured content governance, explicit accessibility and readability standards, and predefined acceptability and qualification thresholds, the protocol offers a transparent and user-centered development process [33,38,90]. All modifications will be documented in a design-decision log to ensure transparency and traceability of development decisions.

### Beta Testing With Naive Users (Future Study)

Beta testing will be conducted as a future study to evaluate the usability, accessibility, and comprehensibility of the DA among naive users—older adults with arthritis and clinicians who did not participate in its development. Consistent with IPDAS and DEVELOPTOOLS checklist [33], this stage will assess real-world navigation patterns, identify usability and literacy-related barriers, and determine whether the DA functions effectively across diverse levels of technology experience and health literacy. A purposive sample of naive users will be recruited to ensure variation in age, gender, arthritis type, cannabis experience, and literacy or technology proficiency, with no advisory-board or alpha-testing participants included. Progression from beta testing to pilot testing will be guided by predefined acceptance thresholds: Patient Education Materials Assessment Tool understandability ≥70% (indicating adequate clarity and ease of understanding), full compliance with the Web Content Accessibility Guidelines (representing the minimum internationally accepted standard for digital accessibility), successful independent navigation of the DA by at least 75% of the sample that will be recruited for beta-testing in the future. and correct interpretation of at least 80% of icons and visual symbols used within the DA [85]. Participants will complete tasks simulating real-world decision-making (eg, information seeking, values clarification, option evaluation) while engaging in a think-aloud exercise to capture navigation challenges and comprehension issues. Usability and comprehension will be measured through structured observation, brief comprehension questions, and the System Usability Scale [91]. Qualitative and quantitative analyses will identify readability or usability barriers to inform the iterative refinement of the prototype before pilot testing. A separate protocol manuscript will describe the full beta-testing methodology, pilot-testing procedures, and implementation-evaluation plan to ensure a rigorous, staged, user-centered evaluation aligned with best practices in DA development.

### Ethical Considerations

This study received ethical approval from the University of Alberta Health Research Ethics Board – Health Panel (Pro00133420) and is registered on the Open Science Framework . All participants will provide informed verbal consent, including explicit consent for audio recording, prior to beginning the interview. Because the interviews explore cannabis decision-making among older adults—a topic that may involve stigma, sensitive disclosures, or safety-related behaviors—additional safeguards have been incorporated [92]. The interviewer will use nonjudgmental, supportive communication techniques and will avoid probing for details related to illicit procurement or illegal activity, while informing participants that confidentiality is upheld within legal and institutional limits [93]. Any disclosure suggesting imminent risk of harm to self or others will be managed according to mandatory institutional duty-to-report procedures [92].

Participants will receive a CAD $30 (approximately US $22) honorarium per interview as compensation for their time, following ethical guidance on avoiding coercion while maintaining fairness [94]. Remote interviews conducted via encrypted institutional Zoom or telephone will follow privacy safeguards for virtual qualitative research, including confirming that participants are in a private location and ensuring that only audio—not video—is recorded [18,95,96]. Alternative modalities (eg, telephone or, when feasible, in-person interviews) will be offered if privacy cannot be adequately maintained. The interviewer will conduct sessions from secure, private environments to ensure reciprocal confidentiality. To safeguard participant privacy, all identifiable data—including audio recordings, transcripts, and field notes—will be systematically deidentified before analysis. Identifiable information and reidentification keys will be stored separately from deidentified transcripts on encrypted, password-protected institutional servers with restricted access for authorized research personnel only. Deidentified data will be retained for 5 years and then securely deleted. Audio recordings will be transcribed verbatim and deleted within 6 months following verification to minimize identifiable data retention. Any adverse events or unintended consequences that arise during interviews or prototype testing will be documented, reviewed, and, when necessary, reported to the Health Research Ethics Board. Because interview conversations may elicit emotional discomfort related to chronic pain, stigma, or treatment decision-making, the interviewer will offer breaks or pause or discontinue the interview upon the participant’s request. Researchers will be ready with information if participants request a resource sheet outlining local arthritis supports, pain management services, and mental health resources; referral pathways will also be available upon request. All participants will receive research team contact information for any follow-up concerns or questions. These integrated safeguards uphold ethical standards and protect participant autonomy and confidentiality.

## Results

### Phase 1: Preliminary Work

#### Phase 1a: Assembling the Advisory Board

Phase 1a is completed as the advisory board is assembled, and it includes 1 rheumatologist (EY), 1 pharmacist (CAS), 1 clinical and experimental science researcher (TKLK), 1 patient-oriented research expert (YNA), 1 graduate student researcher trained in qualitative research (HATA), and 1 patient living with arthritis. In alignment with the IPDAS development process, the advisory board collaboratively defined the scope of the DA. Following IPDAS guidance to clarify the target decision, user population, and context, the board identified the decision as preference-sensitive, involving trade-offs between potential benefits, risks, and uncertainties. Accordingly, the DA was scoped to assist patients and clinicians in engaging in SDM regarding the use of cannabis for symptom management.

#### Phase 1b: Scoping Reviews

Phase 1b is completed. Two scoping reviews were conducted to inform the development of the DA. The first review examined the development and evaluation processes of DSIs in rheumatology [38], identifying 36 DSIs classified as educational tools, DAs, or adaptive conjoint analysis aids. Development was primarily based on frameworks, such as the IPDAS (53%) and the ODSF (16%). Common evaluation measures included the IPDAS qualifying criteria and acceptability assessments. Decision tools varied in format (eg, paper based, web based, interactive) and timing of use (before, during, or after consultations). The second scoping review investigated evidence for cannabis-based medicines in osteoarthritis, revealing mixed evidence with only 60% of studies reporting improvements in pain [58]. Larger studies with longer exposure durations showed no significant benefits. The review highlighted limited high-quality evidence and the need for tailored information addressing patient concerns about safety, efficacy, and potential interactions. Based on the findings from both reviews, we decided to follow established standards (ODSF and IPDAS Development Process Model) emphasizing user-centered methods. These reviews provided actionable insights for developing DSIs tailored to rheumatology patients’ needs regarding cannabis use decisions.

### Phases 2 and 3: Two Qualitative Studies

Phases 2 and 3 are currently being carried out. Recruitment started in January 2024. As of November 2025, we have recruited 10 patients for phase 2, where the majority self-identified as White, over 60 years of age, and with arthritis. For phase 3, we recruited 7 clinicians, including pharmacists, rheumatologists, and nurses with over 5 years of experience as clinicians. We expect to finalize data collection and analysis by early 2026.

### Phase 4: Developing the DA

Phase 4 results will include developing the first draft of the prototype and evaluating its acceptability and qualification as a DA by the advisory board. To date, this phase has not been initiated as it depends on the completion of phases 2 and 3.

## Discussion

### Principal Findings

DAs have been widely endorsed by professional rheumatology and patient care associations as an intervention to improve the quality of health care delivery, with SDM now considered a gold standard in clinical practice [72-74].

Prior work has demonstrated that the suboptimal reporting of the development process of DAs can undermine their later implementation. To mitigate this risk, we adopt established IPDAS-aligned development practices and commit to comprehensive, transparent reporting throughout all phases, consistent with IPDAS guidance that the development phase warrants comprehensive reporting similar to that of evaluation [33,38,90]. The findings from phase 1 and preliminary findings from ongoing qualitative studies of phases 2 and 3 highlight a clear need for informational support to assist decision-making in this context. Both patients and clinicians expressed uncertainty and a desire for credible, accessible information to guide medical cannabis decisions. This reinforces the relevance of our project to tailor a DA that addresses decisional needs specific to patients living with arthritis and clinicians treating arthritis.

This protocol outlines a systematic, multistage approach for the development and evaluation of a theory-informed, evidence-based DA to facilitate SDM regarding medical cannabis use for arthritis-related pain. Our proposed intervention integrates theoretical guidance (ODSF and IPDAS) together with empirical evidence [qualitative studies exploring decisional needs] to produce a DA prototype tailored to arthritis care practice. To our knowledge, this will be the first study to apply a transparent, structured methodology for developing a cannabis-specific DA for individuals living with arthritis.

If successful, this work may facilitate better clinical conversations and alignment of the decision with patient values, while laying the groundwork for broader development and implementation of DAs. We anticipate that such an intervention will improve decision quality, promote patient autonomy, and positively impact patient outcomes, including satisfaction with the decision and quality of life. Furthermore, this protocol contributes to the evolving literature on SDM in arthritis care and expands the evidence base for decision-making support in the context of medical cannabis use.

### Strengths and Limitations

A major strength of this protocol lies in its use of the ODSF, the IPDAS development model, and user-centeredness principles as the foundation guiding the development process [30,41]. The use of ODSF facilitates the identification of decisional needs, while IPDAS provides a structured, evidence-based framework for study procedures and reporting. Together, these models—alongside user-centered design—maximize intended users’ input and enhance the potential usability of the DA [97,98].

The limitations of this study protocol include the fact that the qualitative findings are mostly subjective, and generalizability could be limited. We will try to mitigate this issue by providing a thick description of the findings, setting, and participants. Also, most development decisions are at the discretion of the advisory board, depending on their interpretation of the findings. Therefore, we will transparently report the decisions made by the advisory board with a justification for each decision. Limiting think-aloud alpha testing to advisory board members and other participants involved in DA development may introduce a positive feedback bias. However, such bias is appropriate and expected during alpha-stage refinement, where the goal is to strengthen conceptual clarity and structure before exposing an immature prototype to naive users. Despite these limitations, we are confident that these methods, which are recommended by the IPDAS [33,38] and used by other researchers in the field, together with the expertise of the advisory board members, will lead to quality DA with a high chance of usability and implementation.

## Supplementary material

10.2196/76237 Multimedia Appendix 1 Phases of developing the decision aid.

10.2196/76237 Multimedia Appendix 2 Table of summary of procedures for each phase.

10.2196/76237 Multimedia Appendix 3 Participant flowchart.

10.2196/76237 Multimedia Appendix 4 Interview guide—patients.

10.2196/76237 Multimedia Appendix 5 Interview guide—clinicians.

10.2196/76237 Multimedia Appendix 6 International Patient Decision Aids Standards (IPDAS) qualifying criteria.

10.2196/76237 Multimedia Appendix 7 Acceptability scale.

## Abbreviations

CRA: Canadian Rheumatology Association
DA: decision aid
DSI: decision support intervention
GRADE: Grading of Recommendations Assessment, Development, and Evaluation
IPDAS: International Patient Decision Aids Standards
IPDAS: International Patient Decision Aids Standards
ODSF: Ottawa Decision Support Framework
PRISMA-ScR: Preferred Reporting Items for Systematic reviews and Meta-Analyses extension for Scoping Reviews
RTA: reflexive thematic analysis
SDM: shared decision-making
