# Supplementary material for: Development of a Decision Aid to Support Shared Decision-Making on Cannabis Use for Arthritis: Protocol for a Multiphase Study
Source: JMIR Res Protoc. 2026 Mar 30;15:e76237. doi: 10.2196/76237 (PMC13035037; doi:10.2196/76237)
Supplement: Multimedia Appendix 2 [file resprot-v15-e76237-s002.docx]

**Table (1): Development Phases of DA***

| **Phase** | **Description** | **Completed/ not completed** |
| --- | --- | --- |
| **Phase I: Preliminary Work** | | |
| 1-Assembling the team | A multidisciplinary formal advisory team consisting of experts (rheumatologists, pharmacists, clinical and translational scientists, and patient-oriented research scholars) | Completed |
|  |  |  |
| 2-Defining the scope and purpose of the DA | The advisory board defined the DA:  Scope: to address cannabis use among patients with arthritis  Purpose: to facilitate shared decision-making between the patient and clinician | Completed |
|  |  |  |
| 3-Reviewing and synthesizing the evidence | The assembled team worked with others to review and synthesize the evidence of:   1. DSIs in rheumatology 2. Cannabis-based medicines in osteoarthritis | Completed |
| **Phase II and Phase III: Understanding the user** | |  |
| 1-Involving potential users (arthritis patients and clinicians) in the steps taken to understand the users' needs | The formal qualitative needs assessment will be conducted with patients and clinicians using semi-structured interviews. | In progress |
|  |  |  |
| 2-Inviting potential users (arthritis patients and clinicians) to be part of the advisory board | DSI users who become members of the advisory board will have some level of decisional authority with other members to determine the final version of the DSI. | in progress |
| **Phase IVa: Developing the DSI** | |  |
| Based on the findings of Phases I, II, and III, the DA will be developed | Integrating evidence from the scoping review and qualitative studies, together with advisory board insights | Not initiated yet |
|  |  |  |
| **Phase IVb: Alpha-testing** | | |
| Involving patients and clinicians who participated in phases II and III | Advisory board meetings will take place with patients and clinicians to review the draft design and content before finalizing the prototype. | Not initiated yet |
|  | Advisory board member with patients and clinicians who participated in the qualitative studies  will provide their opinions using think-aloud cognitive methods. |  |
|  |  |  |
|  | The advisory board will discuss feedback from potential users, incorporate it, and then collect feedback again to reach a final prototype version |  |
|  | Changes from one version of a prototype to another will be reported  Decisions made will be listed with a justification for each decision. |  |
| **Future Studies: Beta-testing)** | We will conduct quasi-pre-post studies to measure knowledge and decisional conflict with naïve users before and after using the DA to evaluate it before pilot testing. | Not initiated yet |

*Based on the IPDAS Model development process and DEVELOPTOOLS Reporting Checklist [33]
