# Supplementary material for: Development of a Decision Aid to Support Shared Decision-Making on Cannabis Use for Arthritis: Protocol for a Multiphase Study
Source: JMIR Res Protoc. 2026 Mar 30;15:e76237. doi: 10.2196/76237 (PMC13035037; doi:10.2196/76237)
Supplement: Multimedia Appendix 3 [file resprot-v15-e76237-s003.docx]

**Figure (2):** Participant flow
