# Supplementary material for: Development of a Decision Aid to Support Shared Decision-Making on Cannabis Use for Arthritis: Protocol for a Multiphase Study
Source: JMIR Res Protoc. 2026 Mar 30;15:e76237. doi: 10.2196/76237 (PMC13035037; doi:10.2196/76237)
Supplement: Multimedia Appendix 4 [file resprot-v15-e76237-s004.docx]

## Patient Interview guide^1^

Preamble: Thank you for agreeing to participate in this interview. I am interested in learning about what you need to make a decision (with your clinician if you are given the opportunity) about cannabis use to relieve your rheumatology symptoms. The interview should take about 40min.

What questions do you have before we begin the interview? {interviewer responds to questions}

**Interviewer Initials**: ____ **Date**: ____(dd) /____ (mm)/_____(yy)

**Participant ID number**: **___________________** **Interview #: _______**

**1.** **Demographics**

● Your present age: ___________

● Highest Level of Education: Less than High School, High School, College Certificate, University/College Degree, Post Graduate Training

● How many medications do you take every day? ________ Medications

● In general, would you say your health is: Excellent, Very Good, Good, Fair, or Poor?

● In specific, would you say your rheumatology management is: Excellent, Very Good, Good, Fair, or Poor?

● Do you have insurance that helps to pay for your medications? Yes/No

2. Regarding Cannabis current use. Are you….

- A current cannabis user
- A past cannabis user
- Interested in using cannabis in the future
- May consider using cannabis in the future

All of the information we collect in this voluntary interview will be kept confidential. We’d like your help. It will take about 40 minutes.

I’m going to give you some examples of health decisions some people with rheumatology face. For example, some people need to make:

Decisions about cannabis use to relieve rheumatologic symptoms.

Or

Decisions about treatment options to relieve rheumatologic symptoms.

**DECISION**

1. At this time, what do you think are the most important decisions people with rheumatologic symptoms face?

2. A. Let’s focus on one particular decision that is most important and difficult for you to make relevant to your rheumatology disease. Which one would you choose?

2. B. Let’s focus on another particular decision, the decision is about whether to use cannabis for rheumatologic symptoms or not.

| 3. **Let’s talk about the difficulty of making this decision about using cannabis. How do you feel when making this decision?** | ***[Probe behavioural manifestations of decisional conflict]***  **Do you feel:**  □ unsure about what to do?  □ worried about what could go wrong  □ distressed or upset  □ constantly thinking about the decision  □ wavering between choices or changing their mind  □ delaying the decision  □ questioning what is important to them  □ feeling physically stressed, tense muscles, racing heartbeat, difficulty sleeping] |
| --- | --- |
| 4. **What things make the decision difficult for you?** | ***[Probe factors contributing to decisional conflict]***  **Are you:**  □ Lacking information about options, benefits, risks  □ Lacking information on the chances of benefits and harms  □ Confused about information overload  □ Unclear about what is important to them  □ Feeling unsupported in decision making  □ Feeling pressure from others  □ Lacking motivation or not feeling ready to make a decision  □ Lacking the ability or skill to make a decision |

5. Thinking about this decision, what are the options that you have?

6. What do you see as the main advantages/benefits and disadvantages/risks of the options? [INSERT BELOW USE BACK OF PAGE FOR MORE COMMENTS]

| Option | Advantages/Benefits | Disadvantages/Risks |
| --- | --- | --- |
| 1. Using cannabis to relieve symptoms |  |  |
|  |  |  |
|  |  |  |
|  |  |  |
| 2. Not using cannabis to relieve symptoms |  |  |
|  |  |  |
|  |  |  |
|  |  |  |
|  |  |  |
|  |  |  |
|  |  |  |

| 7. Who else may be involved in making the decision of using cannabis with you? | ***[Probe role in decision making:]***  **Do they usually:**  □ Make the decision for you  □ Share the decision with you  □ Provide support or advice for you to make the decision on your own |
| --- | --- |

|  |  |
| --- | --- |
|  |  |

9. What would help you to make this decision?

10. What will hinder you (get in the way of) making this decision?

11. Is there anything else that would help overcome these barriers to decision-making?

12. I will list possible ways to help some people with a decision, please tell me why each one might or might not be useful to you.

| □ Counseling from a health practitioner 🡪 | IF YES, specify what types of counselling and/or how it would be useful. |
| --- | --- |
| □ Discussion groups of people facing the same decision 🡪 | IF YES, specify what type of organization or group and/or how it would be useful |
| □ Information materials | IF YES, specify the content and/or how it would be useful  □ Health condition  □ Options  □ Benefits  □ Risks  □ Probabilities of benefits/risks  □ Help considering the personal importance of benefits versus risks  □ Guidance in the steps of deliberation and communication  □ Other, specify |
|  | IF YES, specify the format and/or how it would be useful   - Digital (website or app) - Non-digital (booklet or brochure) |

13. Is there anything else that would help better support you in decision-making regarding cannabis use to relieve rheumatologic symptoms?

**CHARACTERISTICS OF PATIENT GROUP**

14. **Gender**

□ Male

□ Female

□ Other/non-specified

□ Prefer not to answer

**16.** **What is the highest grade or level of education you completed?**

□ Less than grade 9

□ Some high school

□ High school diploma

□ Trade certificate/diploma

□ Some college

□ College diploma/degree

□ Some university

□ University undergraduate degree

□ University graduate degree (Masters and PhD)

**17.** **Duration of experience with a health problem**

[THANK RESPONDENT]

**^1^** Jacobsen MJ, O’Connor AM, Stacey D. Decisional needs assessment in populations: a workbook for assessing patients’ and practitioners’ decision making needs. Ottawa, ON, Canada: University of Ottawa Google Scholar. 2013.
