# Supplementary material for: Development of a Decision Aid to Support Shared Decision-Making on Cannabis Use for Arthritis: Protocol for a Multiphase Study
Source: JMIR Res Protoc. 2026 Mar 30;15:e76237. doi: 10.2196/76237 (PMC13035037; doi:10.2196/76237)
Supplement: Multimedia Appendix 5 [file resprot-v15-e76237-s005.docx]

## Clinician Interview guide^1^

Preamble: Thank you for agreeing to participate in this interview. I am interested in learning about what you need to decide whether you should use cannabis for your rheumatologic symptoms or not. The interview should take about 40min.

What questions do you have before we begin the interview? {interviewer responds to questions}

**Interviewer Initials**:____ **Date**: ____(dd) /____ (mm)/_____(yy)

**Participant ID number**: **___________________** **Interview #: _______**

**Age: ……………………….**

**Years of practice: …………………...**

**Setting of practice:………………………………**

**About your practice**

Tell me about your practice

● How many patients with rheumatologic condition(s) do you see every day?

● How often do you get involved in treatment management for patients with rheumatologic conditions?

**Perspectives about information provided on cannabis**

● How do you provide information about cannabis to your patients?

● How important is it for you to provide information about cannabis?

● How has providing or not providing information to the patients about cannabis impacted your practice?

**Regarding Cannabis use:**

- Have you ever been in a situation where you needed to decide with the patient whether to use cannabis or not? If yes, approximately how many times during the past month?
- Why are you interested in decision making regarding cannabis use?

**Your interactions when discussing cannabis use with patients**

● How do you decide which patients require cannabis use?

● Imagine I am a patient diagnosed with rheumatologic disease who is suffering from relevant symptoms. I asked you whether I should use cannabis or not, talk me through how you would go to help me make a decision.

● Do eligible patients (i.e. diagnosed with rheumatology disease and want to know about cannabis) request information to decide whether to use cannabis or not? Why or why not?

o How receptive are patients to using cannabis?

o What else would you like to tell me about cannabis?

**DECISION**

1. What decisions do patients with rheumatology have to make in your practice?

2. A. Let’s focus on one particular decision. One that is most important and difficult for patients to make regarding cannabis use. Which one would you choose?

2. B. Let’s focus on another particular decision, the decision about whether patients would take cannabis to relieve their symptoms.

| 3. **Let’s talk about the difficulty people have making this decision about cannabis use. How do you think patients feel when making this decision? (What could be your description of their feelings towards this decision?)** | ***[Probe behavioural manifestations of decisional conflict]***  **Do they feel:**  □ unsure about what to do?  □ worried about what could go wrong  □ distressed or upset  □ constantly thinking about the decision  □ wavering between choices or changing their mind  □ delaying the decision  □ questioning what is important to them  □ feeling physically stressed, tense muscles, racing heartbeat, difficulty sleeping] |
| --- | --- |
| 4. **What things make the decision difficult for patients?** | ***[Probe factors contributing to decisional conflict]***  **Are patients:**  □ Lacking information about options, benefits, and risks  □ Lacking information on the chances of benefits and harms  □ Confused about information overload  □ Unclear about what is important to them  □ Feeling unsupported in decision making  □ Feeling pressure from others  □ Lacking motivation or not feeling ready to make a decision  □ Lacking the ability or skill to make a decision |

5. What do you see as the main options patients have?

6. What do you see as the main advantages/benefits and disadvantages/risks of the options? [INSERT BELOW USE BACK OF PAGE FOR MORE COMMENTS]

| Option | Advantages/Benefits | Disadvantages/Risks |
| --- | --- | --- |
| 1. Using cannabis to relieve symptoms |  |  |
|  |  |  |
|  |  |  |
|  |  |  |
| 2. Not using cannabis to relieve symptoms |  |  |
|  |  |  |
|  |  |  |
|  |  |  |

| 7. What is your usual role in making this decision? | ***[Probe role in decision-making :]***  **Do they usually:**  □ Make the decision for you  □ Share the decision with you  □ Provide support or advice for you to make the decision on your own |
| --- | --- |

8. What factors make it difficult for you to support your patients’ decision-making regarding whether to use medical cannabis or not?

9. What factors make it easier for you to support your patients’ decision-making whether to use medical cannabis or not?

10. Who else besides yourself and the patient is usually involved in making this decision?

***[Probe:]***

- spouse
- family
- friend
- health care provider
- other, specify

11. What is their usual role in making this decision (i.e. the person mentioned above)?

***[Probe role:]***

**Do they usually:**

Make the decision for the patients

Share the decision with the patients

Provide support or advice for patients to make the decision on their own

Don’t know

Other, specify

12. How does the patient usually go about making such a decision?

***[Probe decision-making behaviour:]***

**Do they:**

Get information on options

Get information on the chances of benefits and risks

Consider the personal importance of the benefits and risks

Get information on how others go about deciding

Get support from others

Find ways to handle pressure

13. What would help patients to make this decision?

14. What will hinder patients (get in the way of) making this decision?

15. Is there anything else that would help overcome barriers to decision-making?

16. I will list possible ways to help some people with a decision. Please tell me why each one might or might not be useful to patients.

| □ Counseling from a health practitioner 🡪 | IF YES, specify what types of counselling and/or how it would be useful. |
| --- | --- |
| □ Discussion groups of people facing the same decision 🡪 | IF YES, specify what type of organization or groups and/or how it would be useful |
| □ Information materials | IF YES, specify the content and/or how it would be useful  □ Health condition  □ Options  □ Benefits  □ Risks  □ Probabilities of benefits/risks  □ Help considering the personal importance of benefits versus risks  □ Guidance in the steps of deliberation and communication  □ Other, specify |
|  | IF YES, specify a format and/or how it would be useful  □   - Digital (website or app) - Non-digital (booklet or brochure) |

17. Is there anything else that would help you to do a better job supporting your patients’ decision-making?

**CHARACTERISTICS OF CLINICIAN**

9. **Gender**

□ Male

□ Female

□ Other/not specified

□ Prefer not to answer

[THANK RESPONDENT]

**^1^** Jacobsen MJ, O’Connor AM, Stacey D. Decisional needs assessment in populations: a workbook for assessing patients’ and practitioners’ decision making needs. Ottawa, ON, Canada: University of Ottawa Google Scholar. 2013.
