# Supplementary material for: Development of a Decision Aid to Support Shared Decision-Making on Cannabis Use for Arthritis: Protocol for a Multiphase Study
Source: JMIR Res Protoc. 2026 Mar 30;15:e76237. doi: 10.2196/76237 (PMC13035037; doi:10.2196/76237)
Supplement: Multimedia Appendix 6 [file resprot-v15-e76237-s006.docx]

**Qualifying Criteria for a Patient Decision Aid***Downloaded from the* [*Ottawa Hospital Research Institute website*](https://decisionaid.ohri.ca/AZinvent.php) *based on the IPDAS Criteria^(1)^*

# Introduction:

[The Ottawa Hospital Research Institute (OHRI)](https://decisionaid.ohri.ca/ipdas.html) applies the [IPDAS (International Patient Decision Aid Standards)](https://pubmed.ncbi.nlm.nih.gov/23963501/) ^(1)^ criteria to systematically evaluate decision aids, ensuring they meet essential quality benchmarks. The assessment focuses on two key areas:

1. Defining a Patient Decision Aid – The decision aid must satisfy [seven core criteria](https://decisionaid.ohri.ca/AZsumm.php), such as explicitly stating the decision to be made, identifying the target audience, listing available options, and providing information on the benefits and risks of each option.
2. Reducing the Risk of Bias – To promote fair and balanced decision-making, the decision aid is evaluated against [eight additional criteria](https://decisionaid.ohri.ca/AZsumm.php) that address issues like presenting options with equal detail, reporting funding sources, and providing references of the evidence.

Each decision aid is reviewed against these standards, and the OHRI assigns a rating based on how many of the seven core and eight bias-reduction criteria it meets. This systematic evaluation helps ensure that decision aids are transparent and balanced and support informed choices in healthcare.

# Objective

We present the OHRI-published criteria (as a structured framework) to help you evaluate the cannabis decision aid, determining whether it meets the standards to be classified as a patient decision aid and whether it effectively reduces the risk of bias in decision-making.

# Procedure:

1. Heba Aref will send you the latest draft of the cannabis decision aid together with the qualifying criteria.
2. Please review the decision aid and answer the questions in the table on the following page.
   1. The estimated time to do this should be no more than 20 minutes?
   2. Complete this by July 8^th^, 2025References

*^(1)^*Joseph-Williams N, Newcombe R, Politi M, et al. Toward Minimum Standards for Certifying Patient Decision Aids: A Modified Delphi Consensus Process. Medical Decision Making. 2013;34(6):699-710. doi:10.1177/0272989X13501721.

**Decision aid qualifying criteria and criteria to lower the risk of making a biased decision based ***

| **Criteria to be defined as a patient decision aid** | **Answer** |
| --- | --- |
| Does the decision aid satisfy the definition of a patient decision aid? Definition of decision aids: interventions designed to help people make specific, deliberative choices. They make the decision explicit, providing information on the options and outcomes relevant to a patient's health status and clarifying personal values. They are intended as adjuncts to counselling. (adapted from [Ohri](https://decisionaid.ohri.ca/cochinvent.php)) | Yes/No |
| The decision aid describes the condition (health or other) related to the decision. | Yes/No |
| The decision aid describes the decision that needs to be considered (the index decision). | Yes/No |
| The decision aid identifies the target audience. | Yes/No |
| The decision aid lists the options (healthcare or other). | Yes/No |
| The decision aid has information about the positive features of the options (e.g. benefits, advantages). | Yes/No |
| The decision aid provides information about the options' negative features (e.g., harms, side effects, and disadvantages). | Yes/No |
| The decision aid helps patients clarify their values for outcomes of options by: a) asking people to think about which positive and negative features of the options matter most to them AND/OR b) describing each option to help patients imagine the physical, social, and /or psychological effect. | Yes/No |
| **Criteria to lower the risk of making a biased decision** | **Answer** |
| • The decision aid makes it possible to compare the positive and negative features of the available options. | Yes/No |
| • The decision aid shows the negative and positive features of the options with equal detail. | Yes/No |
| • The decision aid compares probabilities (e.g. chance of a disease, benefit, harm, or side effect) of options using the same denominator. | Yes/No |
| • The decision aid (or available technical documents) reports funding sources for development. | Yes/No |
| • The decision aid reports whether authors of the decision aid or their affiliations stand to gain or lose by choices people make after using it. | Yes/No |
| • The decision aid includes authors'/developers' credentials or qualifications. | Yes/No |
| • The decision aid reports the date when it was last updated. | Yes/No |
| • The decision aid (or available technical document) reports readability levels. | Yes/No |
| • The decision aid provides references to the scientific evidence used. | Yes/No |

*Downloaded from [The Ottawa Hospital Research Institute patient aids research group](https://decisionaid.ohri.ca/AZsumm.php?ID=2076) based on International Patient Decision Aid Standards ([IPDAS)](http://ipdas.ohri.ca/) criteria ^1^

^1^ Joseph-Williams N, Newcombe R, Politi M, et al. Toward Minimum Standards for Certifying Patient Decision Aids: A Modified Delphi Consensus Process. Medical Decision Making. 2013;34(6):699-710. [doi:10.1177/0272989X13501721](https://journals.sagepub.com/doi/10.1177/0272989X13501721)
