# Supplementary material for: Development of a Decision Aid to Support Shared Decision-Making on Cannabis Use for Arthritis: Protocol for a Multiphase Study
Source: JMIR Res Protoc. 2026 Mar 30;15:e76237. doi: 10.2196/76237 (PMC13035037; doi:10.2196/76237)
Supplement: Multimedia Appendix 7 [file resprot-v15-e76237-s007.docx]

**Acceptability of the Cannabis Decision Aid
*For Clinicians and Advisory Committee Members***

# Introduction

This Acceptability measure is developed to be used during the development process and early evaluation of a decision aid. Acceptability (of a decision aid) refers to ratings regarding the comprehensibility of components of a decision aid and overall suitability for decision making.

# Objective

The purpose of this assessment is to evaluate the acceptability of the cannabis decision aid for decision aid advisory committee members and clinicians working with patients considering cannabis for arthritis symptom management. Your participation is valuable as it will help refine the decision aid to ensure it effectively meets patient needs to make informed decisions.

# Procedure

1. Research assistant Heba Aref sent you the cannabis decision aid together with this acceptability measure.
2. You will answer the provided survey and open-ended questions about the cannabis decision aid.

# Expected Output

This acceptability measure will assess the following:
- Clarity and comprehensibility of information.
- Balance and neutrality in presenting cannabis as an option.
- Appropriateness of the amount of information provided.
- Overall usefulness of the decision aid in assisting with decision-making.

# Citation

O'Connor AM & Cranney A. User Manual – Acceptability [document on the Internet]. Ottawa: Ottawa Hospital Research Institute; © 1996 [modified 2002; cited 2025 05 22]. Available from: <http://decisionaid.ohri.ca/docs/develop/User_Manuals/UM_Acceptability.pdf>

# Studies that used the Acceptability Tool

1. Man-Son-Hing M, Laupacis A, O'Connor A, Wells G, Lemelin J, Wood W, Dermer M. Warfarin for atrial fibrillation. The patient's perspective. Archives of Internal Medicine 1996; 156: 1841-1848.

2. O'Connor AM, Tugwell P, Wells GA, Elmslie T, Jolly E, Hollingworth G, McPherson R, Bunn H, Graham I, Drake E. A decision aid for women considering hormone therapy after menopause: decision support framework and evaluation. Patient Education and Counseling 1998; 33: 267-279.

3. O'Connor AM, Tugwell P, Wells GA, Elmslie T, Jolly E, Hollingworth G, McPherson R, Drake E, Hopman W, MacKenzie T. Randomized Trial of a Portable, Self-administered Decision Aid for Postmenopausal Women Considering Long-term Preventive Hormone Therapy. Medical Decision Making 1998; 18: 295-303.

4. Drake E, Engler-Todd L, O'Connor AM, Surh L, Hunter A. Development and evaluation of a decision aid about prenatal testing for women of advanced maternal age. Journal of Genetic Counselling 1999; 8: 217-233.

5. Fiset V, O'Connor AM, Evans W, Graham I, DeGrasse C, Logan J. Development and evaluation of a decision aid for patients with stage IV non-small cell lung cancer. Health Expectations 2000; 3: 125-136.

6. Grant FC, Laupacis A, O'Connor AM, Rubens F, Robblee J. Evaluation of a decision aid for patients considering autologous blood donation before open-heart surgery. Canadian Medical Association Journal 2001; 164: 1139-1144.

7. Cranney A, O'Connor AM, Jacobsen MJ, Tugwell P, Adachi JD, Ooi DS, Waldegger L, Goldstein R, Wells GA. Development and pilot testing of a decision aid for postmenopausal women with osteoporosis. Patient Education and Counseling 2002; 47: 245-255.

# My opinions on the decision aid addressing cannabis

**Demographic data:**

**1. Age:**
Please indicate your age (in years): _______

**2. Gender:**
Please select your gender:
☐ Woman
☐ Man
☐ Non-binary / Third gender
☐ Prefer to self-describe: ____________
☐ Prefer not to say

**3. Area of Specialty or Clinical/Academic Practice:**Please specify your primary area(s) of clinical or academic practice (e.g., rheumatology, geriatrics, pharmacy, health policy, medical education, pharmacology, clinical and experimental sciences etc.)**:**

**4. Clinical Community or Professional Group Represented:**
Please indicate the clinician community, professional group, or organization you represent (e.g., Academia, Faculyt of Pharmacy, Faculty of Medicine, Canadian Rheumatology Association, College of Family Physicians, independent practitioner, etc.):

**The following questions ask about your perceptions of the cannabis decision aid. We are interested in your reactions to the decision aid that PhD Candidate Heba Aref sent you.**

By underlining and bolding the appropriate number, please indicate how strongly you agree or disagree with each statement.

| In general: | Strongly Disagree | Disagree | Agree | Strongly Agree |
| --- | --- | --- | --- | --- |
| 1. The cannabis decision aid will be easy for me to use. | 1 | 2 | 4 | 5 |
| 1. The cannabis decision aid is easy for me to understand. | 1 | 2 | 4 | 5 |
| 1. The cannabis decision aid will be easy for me to experiment with before making a final decision to adopt it. | 1 | 2 | 4 | 5 |
| 1. The results of using the cannabis decision aid will be easy to see | 1 | 2 | 4 | 5 |
| 1. This cannabis decision aid is better than how I usually go about helping patients decide about cannabis use. | 1 | 2 | 4 | 5 |
| 1. The cannabis decision aid is compatible with the way I think things should be done. | 1 | 2 | 4 | 5 |
| 1. The use of this cannabis decision aid is more cost effective than my usual approach to helping patients decide about cannabis. | 1 | 2 | 4 | 5 |
| 1. Compared with my usual approach, this cannabis decision aid will result in my patients making more informed decisions. | 1 | 2 | 4 | 5 |
| 1. Using this cannabis decision aid will save me time. | 1 | 2 | 4 | 5 |
| 1. This cannabis decision aid is a reliable method of helping patients make decisions about cannabis. | 1 | 2 | 4 | 5 |
| 1. This cannabis decision aid can be used by the patients themselves on their own. | 1 | 2 | 4 | 5 |
| 1. This cannabis decision aid is suitable for helping patients make value-laden choices. | 1 | 2 | 4 | 5 |
| 1. This cannabis decision aid complements the my usual approach. | 1 | 2 | 4 | 5 |
| 1. Using this cannabis decision aid does not involve making major changes to the way I usually do things | 1 | 2 | 4 | 5 |
| 1. There is a high probability that using this cannabis decision aid may cause/result in more benefit than harm | 1 | 2 | 4 | 5 |

1. What did you like about the decision aid?
2. What suggestions do you have to improve the decision aid?

**Thank you**
